# Uncommon *Salmonella* Infantis Variants with Incomplete Antigenic Formula in the Poultry Food Chain, Italy

**DOI:** 10.3201/eid3004.231074

**Published:** 2024-04

**Authors:** Sara Petrin, Alessia Tiengo, Alessandra Longo, Maddalena Furlan, Elisa Marafin, Paola Zavagnin, Massimiliano Orsini, Carmen Losasso, Lisa Barco

**Affiliations:** National and World Organisation for Animal Health Reference Laboratory for *Salmonella*, Istituto Zooprofilattico Sperimentale delle Venezie, Legnaro, Italy

**Keywords:** *Salmonella enterica*, *Salmonella* Infantis, emerging atypical variants, enteric infections, poultry, Salmonellae, Italy, bacteria, food safety

## Abstract

Uncommon *Salmonella* Infantis variants displaying only flagellar antigens phenotypically showed identical incomplete antigenic formula but differed by molecular serotyping. Although most formed rough colonies, all shared antimicrobial resistances and the presence of *usg* gene with wild-type *Salmonella* Infantis. Moreover, they were undistinguishable wild-type *Salmonella* Infantis by whole-genome sequencing.

The emergence of variants posing threats to human health and animal production characterizes the epidemiology of *Salmonella* (*1*) and also *S. enterica* serovar Infantis (antigenic formula 6,7:r:1,5). Over the past few decades, this serovar has rapidly emerged along the poultry chain; as of 2023, it is the most prevalent serovar isolated from broiler chickens in the European Union and among the 4 most common serovars in humans (*2*,*3*). The European Commission identified *Salmonella* Infantis as a target serovar for which control measures must be implemented if it is isolated in breeding flocks of *Gallus gallus* chickens (*4*). Some of the mandatory control measures implemented in Italy are appropriate health measures applied at farms; eradication or slaughtering of *Salmonella*-positive birds and management of their carcasses in accordance with EC regulation no. 1069/2009; and disposal of eggs produced by *Salmonella* Infantis‒positive groups and additional cleaning and disinfection procedures of the flock environment and facilities. The strict and targeted control measures implemented in case of identification of *Salmonella* Infantis‒positive flocks (*5*) require standardized analytical methods (i.e., ISO 6579:1 for isolation of *Salmonella* and methods based on the Kaufmann-White scheme for serotyping or validated alternative methods) to ensure high quality of surveillance, prompt identification of positive samples, and prompt implementation of eradication measures.

Isolates with an incomplete antigenic formula that carried flagellar antigens typical of *Salmonella* Infantis (r:1,5), but lacked the somatic ones (6,7), have been increasingly isolated from poultry sources in Italy. Laboratories in charge of *Salmonella* controls and surveillance needed to quickly identify and characterize these isolates to ascertain if these atypical variants were *Salmonella* Infantis strains and consequently manage their isolation on farms for breeding *G. gallus* fowl. Our investigation also included strains showing those atypical features isolated from food matrices, to estimate their spread along the poultry chain.

## The Study

We tested a total of 31 *Salmonella* strains that could be ascribed to *Salmonella* Infantis but lacked the expression of the complete antigenic formula from animals (N = 20) and food (N = 11). Those strains were isolated during 2014‒2022 from samples taken in different regions of Italy (Table). We included 1 wild-type *Salmonella* Infantis strain isolated from food as a reference. We serotyped all *Salmonella* isolates by slide agglutination with *Salmonella* antiserum samples (Statens Serum Institut, Copenhagen, Denmark); we assigned antigenic formulas in accordance with ISO 6579:3. If traditional serotyping could not provide conclusive results because the complete antigenic formula was not expressed, we performed molecular serotyping by using an xMAP *Salmonella* serotyping assay (*6*). In particular, a positive match from the somatic antigen with C1 probe (*wzy* gene) is expected in case of wild-type *Salmonella* Infantis isolates. Colony morphology was investigated on agar tryptose solid medium. We assessed antimicrobial susceptibility by MIC by using the broth microdilution method with the Sensititer EUVSEC panel (TREK Diagnostics System; ThermoFisher, https://www.thermofisher.com) and interpreted results in accordance with European Committee on Antibiotic Susceptibility Testing (EUCAST) epidemiologic cutoff values. We selected the *usg* gene (SIN_02055) as a marker gene specific for *Salmonella* Infantis; we used the PCR targeting this gene described by Yang et al. (*7*) as an identification marker to test whether strains belonged to *Salmonella* Infantis serovar. Finally, we performed whole-genome sequencing as described in Petrin et al. (*8*) and used a core genome multilocus sequence typing (cgMLST) scheme approach (*9*) to assess genetic relatedness among the investigated strains and with the wild-type *Salmonella* Infantis strain. We also performed in-silico serotyping as described by Costa et al. (*9*).

**Table:** *Salmonella enterica* serovar Infantis isolates in the poultry food chain, Italy, 2014‒2022*

Isolate ID	Matrix	Source	Year	Location	Antigenic formula			ST
					Seroagglutination†	Molecular serotyping‡	WGS§	
21-153004-9	Meat	Food	2014	Veneto	*S*. -: r: 1,5	*S*. Infantis (C1: r: 1,5)	O-7:r:1,5 (Infantis)	ST32
21-153004-10	Meat	Food	2014	Veneto	*S*. -: r: 1,5	*S*. Infantis (C1: r: 1,5)	O-30:r:1,5 (Gege)	ST32
21-153004-11	Meat	Food	2014	Veneto	*S*. -: r: 1,5	*S*. -: r: 1,5	O-?:r:1,5	ST32
21-153004-12	Sock sample	Animal	2014	Veneto	*S*. -: r: 1,5	*S*. Infantis (C1: r: 1,5)	O-?:r:1,5	ST32
21-153004-13	Sock sample	Animal	2016	Veneto	*S*. -: r: 1,5	*S*. -: r: 1,5	O-?:r:1,5	ST32
21-153004-14	Meat	Food	2016	Veneto	*S*. -: r: 1,5	*S*. -: r: 1,5	O-?:r:1,5	ST32
21-153004-1	Sock sample	Animal	2016	Lombardia	*S*. -: r: 1,5	*S*. Infantis (C1: r: 1,5)	O-30:r:1,5 (Gege)	ST32
21-153014-1	Sock sample	Animal	2017	Lombardia	*S*. -: r: 1,5	*S*. -: r: 1,5	O-?:r:1,5	ST32
21-153014-2	Sock sample	Animal	2017	Lombardia	*S*. -: r: 1,5	*S*. -: r: 1,5	O-?:r:1,5	ST32
21-153014-3	Sock sample	Animal	2017	Molise	*S*. -: r: 1,5	*S*. -: r: 1,5	O-?:r:1,5	ST32
21-153014-4	Sock sample	Animal	2017	Molise	*S*. -: r: 1,5	*S*. -: r: 1,5	O-?:r:1,5	ST32
21-153014-14	Sock sample	Animal	2017	Molise	*S*. -: r: 1,5	*S*. -: r: 1,5	O-?:r:1,5	ST32
21-153004-2	Meat	Food	2018	Veneto	*S*. -: r: 1,5	*S*. -: r: 1,5	O-?:r:-	ST32
21-153004-3	Carcass	Animal	2018	Veneto	*S*. -: r: 1,5	*S*. Infantis (C1: r: 1,5)	O-7:r:1,5 (Infantis)	ST32
21-153014-5	Meat	Animal	2018	Veneto	*S*. -: r: 1,5	*S*. -: r: 1,5	O-?:r:1,5	ST32
21-153014-7	Sock sample	Animal	2018	Veneto	*S*. -: r: 1,5	*S*. -: r: 1,5	O-?:r:1,5	ST32
18-1157-6	Meat	Food	2018	Veneto	*S*. -: r: 1,5	*S*. -: r: 1,5	O-?:r:1,5	ST32
21-153004-4	Sock sample	Animal	2019	Puglia	*S*. -: r: 1,5	*S*. -: r: 1,5	O-?:r:1,5	ST32
21-153004-5	Meat	Food	2019	Veneto	*S*. -: r: 1,5	*S*. -: r: 1,5	O-?:r:1,5	ST32
21-153004-6	Carcass	Animal	2019	Veneto	*S*. -: r: 1,5	*S*. Infantis (C1: r: 1,5)	O-7:r:1,5 (Infantis)	ST32
21-153014-11	Sock sample	Animal	2020	Veneto	*S*. -: r: 1,5	*S*. Infantis (C1: r: 1,5)	O-30:r:1,5 (Gege)	ST32
21-128165	Meat	Food	2021	Friuli Venezia Giulia	*S*. -: r: 1,5	*S*. -: r: 1,5	O-?:r:1,5	ST32
21SAL/2851/1	Sock sample	Animal	2021	Lazio	*S*. -: r: 1,5	*S*. -: r: 1,5	O-?:r:1,5	ST32
21-146541	Sock sample	Animal	2021	Lazio	*S*. -: r: 1,5	*S*. -: r: 1,5	O-?:r:1,5	ST32
21-21379	Sock sample	Animal	2021	Veneto	*S*. -: r: 1,5	*S*. -: r: 1,5	O-?:r:1,5	ST32
21-117758	Meat	Food	2021	Emilia Romagna	*S*. -: r: 1,5	*S*. -: r: 1,5	O-?:r:1,5	ST32
21-117759	Sock sample	Animal	2021	Umbria	*S*. -: r: 1,5	*S*. -: r: 1,5	O-?:r:1,5	ST32
22-16858-1	Meat	Food	2022	Sardegna	*S*. -: r: 1,5	*S*. -: r: 1,5	O-?:r:-	ST32
22-23625-9	Carcass	Animal	2022	Friuli Venezia Giulia	*S*. -: r: 1,5	*S*. Infantis (C1: r: 1,5)	O-30:r:1,5 (Gege)	ST32
22-39728-9	Carcass	Animal	2022	Molise	*S*. -: r: 1,5	*S*. -: r: 1,5	O-?:r:1,5	ST32
22-102991-7	Carcass	Food	2022	Veneto	*S*. -: r: 1,5	*S*. Infantis (C1: r: 1,5)	O-7:r:1,5 (Infantis)	ST5275
22-16858-3	Meat	Animal	2022	Sardegna	*S*. Infantis (6,7: r: 1,5)	*S*. Infantis (C1: r: 1,5)	O-7:r:1,5 (Infantis)	ST32

*Reference isolate showed complete antigenic formula (22-16858-3). ID, identification; ST, sequence type. †As determined by traditional seroagglutination (ISO 6579:3). ‡As determined by molecular serotyping (xMAP *Salmonella* serotyping assay [*6*]). §As determined by whole-genome sequencing.

**Figure 1:**
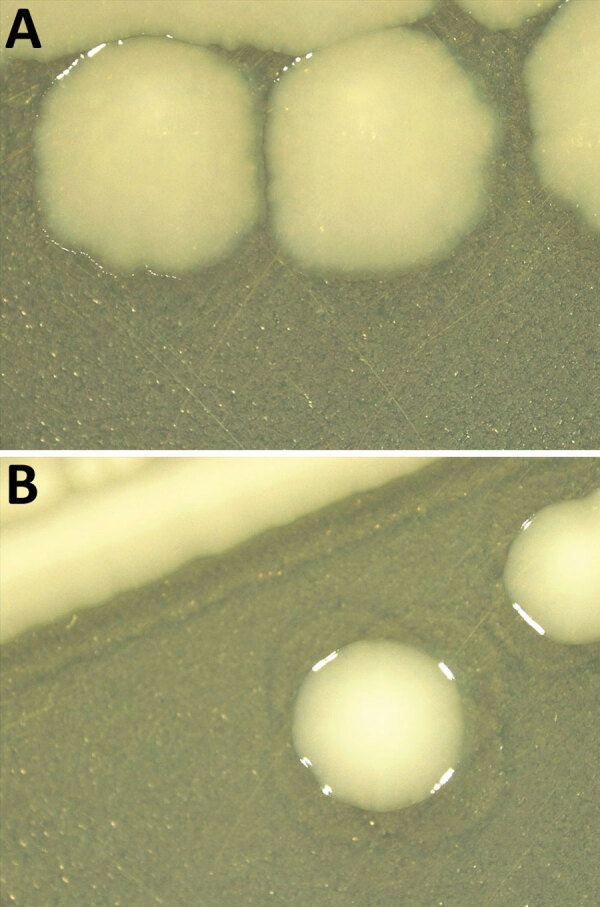
Comparison of colony morphology *Salmonella enterica* serovar Infantis isolates collected in Italy, 2014‒2022. Isolates were grown on agar tryptose solid medium for 24 hours. A) Rough colonies from *Salmonella* Infantis atypical isolates (*S*. -:r:1,5). B) Smooth colonies from wild-type *Salmonella* Infantis isolates (*S*. 6,7:r:1,5).

Thirty-one of 32 strains did not agglutinate with the somatic serum specimens for 6,7 antigens, indicating that their antigenic formula was -:r:1,5 (Table). Molecular serotyping identified a match with the C1 somatic probe in 9 strains. Of those, 3 were isolated from food and 6 from animal sources. For the remaining 22 strains, detection with the C1 somatic probe was negative. The detection of typical *Salmonella* Infantis flagellar antigens was positive for all the tested strains. In silico serotyping did not provide conclusive results on the O-antigen encoding region for 27 isolates; those results support the need for further analyses. Twenty-six strains displayed rough colony morphology (Figure 1), which in many cases contributes to increased sensitivity to immune defense, even if it remains unclear whether these strains are pathogenic (*10*). Most of the selected strains shared a similar resistance pattern to multiple antimicrobial drugs (ampicillin, ciprofloxacin, azithromycin, nalidixic acid, tetracycline, trimethoprim, and sulfamethoxazole) (Appendix Table) typical of the circulating wild-type *Salmonella* Infantis strains spread in broiler populations (*1*). 

**Figure 2:**
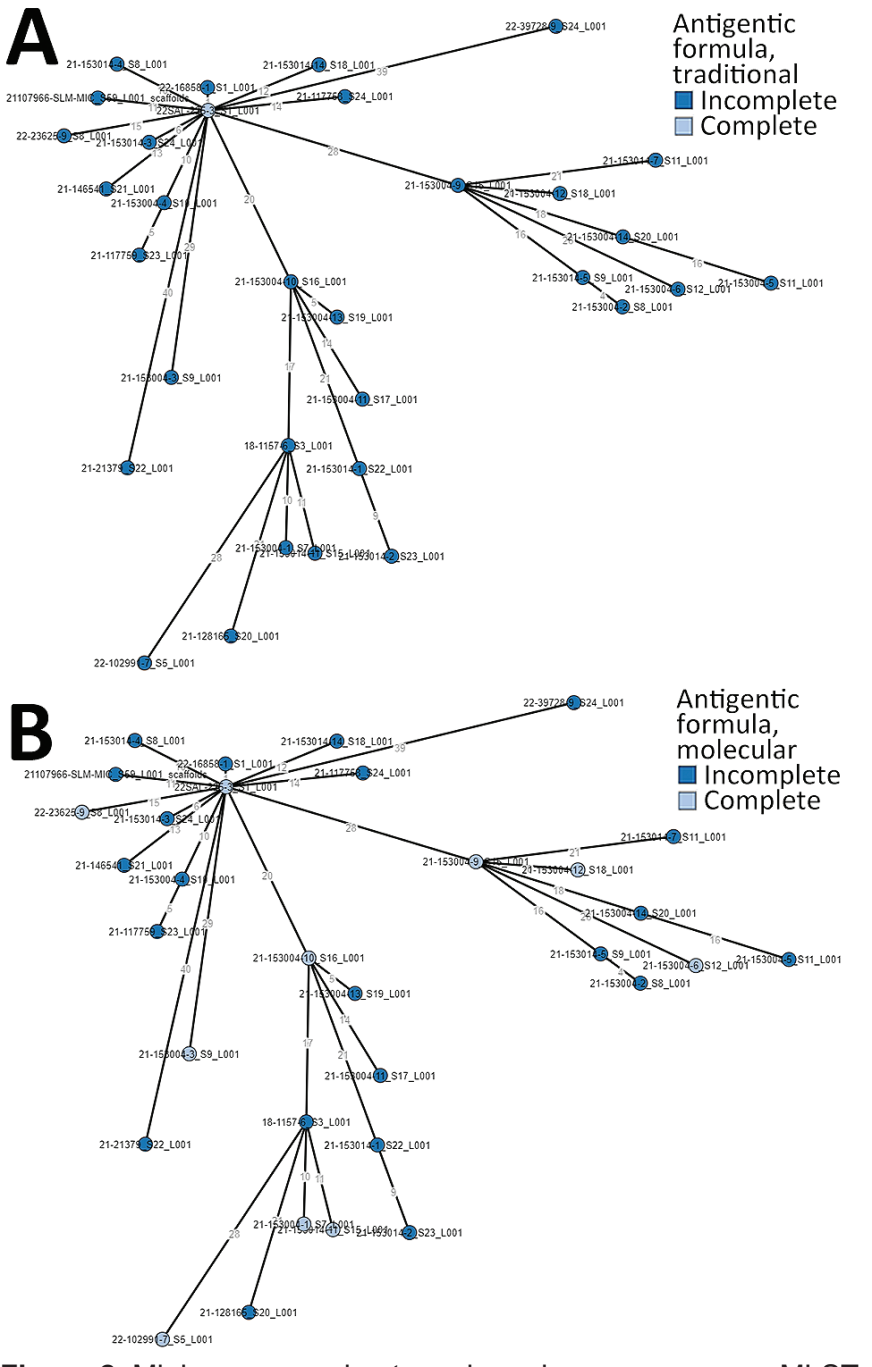
Minimum-spanning trees based on core genome MLST analysis of *Salmonella* *enterica* serovar Infantis strains collected in Italy, 2014‒2022, including atypical isolates (*S*. -:r:1,5). A) Traditional serotyping. B) Molecular serotyping.

All the 31 isolates harboring incomplete antigenic formula, as well as the isolate with the complete antigenic formula, had *usg* gene present in their genomes; the presence of the gene can be considered a promising tool for rapid diagnosis of those atypical strains with incomplete expression of somatic antigens. The investigations carried out by cgMLST to establish the genetic relatedness among isolates did not enable us to identify different *Salmonella* Infantis populations on the basis of their antigenic formula, considering both traditional serotyping (Figure 2, panel A) and molecular serotyping (Figure 2, panel B).

## Conclusions 

Our results describe heterogeneous *Salmonella* Infantis strains widespread in the poultry food chain in Italy. This heterogeneity seems to involve the antigen-coding genetic context that probably would also affect the colony morphology (*11*). It is well known that rough morphology of *Salmonella* isolates originates from deletion or truncations of lipopolysaccharide O-antigen encoding genes (*12*). In our study, however, we could not link the rough phenotype, identified for the atypical strains, to the absence of the *wzy* gene nor to the isolation matrix and source. Further analyses at the genomic level will clarify the deletion/truncation pattern and identify the involved genes; the genetic region encoding for the O-antigen is composed of many different genes (*13*,*14*). 

We found atypical strains that lacked the expression of the complete antigenic formula to be genetically indistinguishable from wild-type *Salmonella* Infantis. We also found a clear indication toward an antimicrobial resistance profile, shared by all the investigated atypical isolates, that provided resistance to multiple antimicrobial compounds commonly described for the wild-type circulating clones of *Salmonella* Infantis (*1*,*15*). This similarity leads to a likely closeness of those somatic variant isolates to the wild-type *Salmonella* Infantis isolates, showing a complete antigenic formula. The close relationships between *Salmonella* Infantis and variant isolates with an incomplete antigenic formula and the reasons for the lack of O-antigen expression should be further investigated. Identifying the atypical strains would pose a diagnostic issue because they would not be recognized as *Salmonella* Infantis by traditional serotyping, which remains by far the most common method used by laboratories in charge of *Salmonella* surveillance. This difficulty in identifying such atypical strains could compromise the prompt recognition of infected poultry flocks and hamper the timely implementation of the targeted control measures required by legislation, which would have serious repercussions on public health.

Appendix Additional information uncommon *Salmonella* Infantis variants with incomplete antigenic formula in the poultry food chain, Italy.
