# Supplementary material for: Uncommon Salmonella Infantis Variants with Incomplete Antigenic Formula in the Poultry Food Chain, Italy
Source: Emerg Infect Dis. 2024 Apr;30(4):795–9. doi: 10.3201/eid3004.231074 (PMC10977818; doi:10.3201/eid3004.231074)
Supplement: Appendix — Additional information uncommon Salmonella Infantis variants with incomplete antigenic formula in the poultry food chain, Italy. [file 23-1074-Techapp-s1.pdf]

Article DOI: http://doi.org/10.3201/eid3004.231074

EID cannot ensure accessibility for supplementary materials supplied by authors. Readers who have difficulty accessing supplementary content should contact the authors for assistance.

Uncommon *Salmonella* Infantis Variants with Incomplete Antigenic Formula in the Poultry Food Chain, Italy

Appendix Table. Susceptibility profile of 32 *Salmonella* isolates in the poultry food chain, Italy*

Isolate ID	AMP	MERO	CIP	AZI	AMI	GEN	TGC	TAZ	FOT	CHL	COL	NAL	TET	TMP	SMX
21–153004–9	**>32 R**	<0,03 S	**0,5 R**	32 NI	<4 S	<0,5 S	2 NI	1 S	0,5 S	**32 R**	<1 NI	**>64 R**	**>32 R**	**>16 R**	>512 NI
21–153004–10	2 S	<0,03 S	**0,5 R**	16 NI	<4 S	<0,5 S	1 NI	1 S	<0,25 S	**32 R**	<1 NI	**>64 R**	**>32 R**	**>16 R**	>512 NI
21–153004–11	4 S	<0,03 S	**0,5 R**	32 NI	<4 S	<0,5 S	0,5 NI	1 S	0,5 S	16 S	<1 NI	**>64 R**	4 S	**>16 R**	64 NI
21–153004–12	**>32 R**	<0,03 S	**0,5 R**	32 NI	<4 S	<0,5 S	2 NI	1 S	<0,25 S	**32 R**	<1 NI	**>64 R**	**>32 R**	**>16 R**	>512 NI
21–153004–13	2 S	<0,03 S	**0,25 R**	16 NI	<4 S	<0,5 S	2 NI	1 S	<0,25 S	16 S	<1 NI	**>64 R**	**>32 R**	**>16 R**	>512 NI
21–153004–14	**>32 R**	<0,03 S	**0,5 R**	32 NI	<4 S	<0,5 S	2 NI	2 S	0,5 S	**32 R**	<1 NI	**>64 R**	**>32 R**	<0,25 S	>512 NI
21–153004–1	**>32 R**	<0,03 S	**0,5 R**	16 NI	<4 S	<0,5 S	0,5 NI	2 S	0,5 S	16 S	<1 NI	**>64 R**	**>32 R**	**>16 R**	>512 NI
21–153014–1	**>32 R**	<0,03 S	**0,25 R**	32 NI	<4 S	<0,5 S	0,5 NI	1 S	0,5 S	**>64 R**	<1 NI	**>64 R**	**>32 R**	**>16 R**	>512 NI
21–153014–2	**>32 R**	<0,03 S	**0,5 R**	32 NI	<4 S	<0,5 S	1 NI	1 S	<0,25 S	**>64 R**	<1 NI	**>64 R**	**>32 R**	<0,25 S	>512 NI
21–153014–3	**>32 R**	<0,03 S	**0,5 R**	32 NI	<4 S	<0,5 S	1 NI	**>8 R**	**>4 R**	16 S	<1 NI	**>64 R**	**>32 R**	**>16 R**	>512 NI
21–153014–4	**>32 R**	<0,03 S	**0,25 R**	32 NI	<4 S	<0,5 S	1 NI	**8 R**	**>4 R**	16 S	<1 NI	**>64 R**	**>32 R**	**>16 R**	64 NI
21–153014–14	**>32 R**	<0,03 S	**0,25 R**	32 NI	<4 S	<0,5 S	1 NI	**>8 R**	**>4 R**	16 S	<1 NI	**>64 R**	**>32 R**	**>16 R**	>512 NI
21–153004–2	2 S	<0,03 S	**0,25 R**	32 NI	<4 S	<0,5 S	1 NI	1 S	0,5 S	16 S	<1 NI	**>64 R**	**>32 R**	**>16 R**	>512 NI
21–153004–3	4 S	<0,03 S	**0,5 R**	16 NI	<4 S	<0,5 S	1 NI	1 S	0,5 S	**32 R**	<1 NI	**>64 R**	**>32 R**	**>16 R**	>512 NI
21–153014–5	4 S	<0,03 S	**0,5 R**	32 NI	<4 S	<0,5 S	2 NI	1 S	1 S	16 S	<1 NI	**>64 R**	**>32 R**	**>16 R**	>512 NI
21–153014–7	2 S	<0,03 S	**0,5 R**	32 NI	<4 S	<0,5 S	1 NI	2 S	0,5 S	**32 R**	<1 NI	**>64 R**	**>32 R**	**>16 R**	>512 NI
18–1157–6	8 S	<0,03 S	**0,25 R**	16 NI	<4 S	<0,5 S	2 NI	1 S	0,5 S	16 S	<1 NI	**>64 R**	**>32 R**	**>16 R**	>512 NI
21–153004–4	**>32 R**	<0,03 S	**0,25 R**	16 NI	<4 S	<0,5 S	1 NI	**8 R**	**>4 R**	16 S	<1 NI	**>64 R**	**>32 R**	**>16 R**	>512 NI
21–153004–5	**>32 R**	<0,03 S	**0,5 R**	32 NI	<4 S	<0,5 S	1 NI	1 S	0,5 S	16 S	<1 NI	**>64 R**	**>32 R**	<0,25 S	>512 NI
21–153004–6	4 S	<0,03 S	**0,25 R**	32 NI	<4 S	<0,5 S	0,5 NI	1 S	0,5 S	16 S	2 NI	**>64 R**	4 S	**>16 R**	64 NI
21–153014–11	2 S	<0,03 S	**0,5 R**	16 NI	<4 S	<0,5 S	1 NI	1 S	<0,25 S	**32 R**	<1 NI	**>64 R**	**>32 R**	**>16 R**	>512 NI
21–128165	4 S	<0,03 S	**0,5 R**	16 NI	<4 S	<0,5 S	1 NI	1 S	0,5 S	16 S	<1 NI	**>64 R**	**>32 R**	**>16 R**	>512 NI
21SAL/2851/1	**>32 R**	<0,03 S	**0,5 R**	16 NI	<4 S	<0,5 S	0,5 NI	**8 R**	**>4 R**	16 S	<1 NI	**>64 R**	**>32 R**	**>16 R**	>512 NI
21–146541	2 S	<0,03 S	**0,5 R**	16 NI	<4 S	<0,5 S	1 NI	1 S	<0,25 S	16 S	<1 NI	**>64 R**	**>32 R**	**>16 R**	>512 NI
21–21379	4 S	<0,03 S	**0,5 R**	16 NI	<4 S	<0,5 S	2 NI	1 S	<0,25 S	16 S	<1 NI	**>64 R**	**>32 R**	**>16 R**	>512 NI
21–117758	**>32 R**	<0,03 S	**0,25 R**	16 NI	<4 S	<0,5 S	1 NI	**8 R**	**>4 R**	16 S	<1 NI	**>64 R**	**>32 R**	**>16 R**	>512 NI
21–117759	2 S	<0,03 S	**0,5 R**	16 NI	<4 S	<0,5 S	0,5 NI	1 S	<0,25 S	**32 R**	<1 NI	**>64 R**	**>32 R**	**>16 R**	>512 NI
22–16858–1	2 S	<0,03 S	**0,25 R**	16 NI	<4 S	<0,5 S	1 NI	1 S	<0,25 S	16 S	<1 NI	**>64 R**	**>32 R**	**>16 R**	>512 NI
22–23625–9	2 S	<0,03 S	**0,25 R**	32 NI	<4 S	<0,5 S	1 NI	1 S	<0,25 S	16 S	<1 NI	**>64 R**	**>32 R**	**>16 R**	>512 NI
22–39728–9	2 S	<0,03 S	**0,25 R**	32 NI	<4 S	<0,5 S	1 NI	1 S	<0,25 S	16 S	<1 NI	**>64 R**	**>32 R**	**>16 R**	>512 NI
22–102991–7	2 S	<0,03 S	**0,25 R**	8 NI	<4 S	<0,5 S	0,5 NI	0,5 S	0,5 S	<8 S	<1 NI	**>64 R**	**>32 R**	**>16 R**	>512 NI
22–16858–3	<1 S	<0,03 S	**0,12 R**	8 NI	<4 S	<0,5 S	0,5 NI	0,5 S	<0,25 S	<8 S	<1 NI	**>64 R**	**>32 R**	**>16 R**	>512 NI
**Total no. resistant isolates (%)**	**13 (40.63)**	0	**32 (100)**	0	0	0	0	**6 (18.75)**	**6 (18.75)**	**10 (31.25)**	0	**32 (100)**	**30 (93.75)**	**29 (90.63)**	0

*Bold text indicates MIC values considered indicative of resistance. AMP, ampicillin; AZI, azithromycin; CHL, chloramphenicol; CIP, ciprofloxacin; COL, colistin; FOT, cefotaxime; GEN, gentamycin; MERO, meropenem; NAL, nalidxic acid; S, susceptible; SMX, sulfamethoxazole; TAZ, ceftazidime; TET, tetracycline; TGC, tigecycline; TMP, trimethoprim.
